# Low-Frequency rTMS Ameliorates Autistic-Like Behaviors in Rats Induced by Neonatal Isolation Through Regulating the Synaptic GABA Transmission

**DOI:** 10.3389/fncel.2018.00046

**Published:** 2018-02-28

**Authors:** Tao Tan, Wei Wang, Haitao Xu, Zhilin Huang, Yu Tian Wang, Zhifang Dong

**Affiliations:** ^1^Ministry of Education Key Laboratory of Child Development and Disorders and Chongqing Key Laboratory of Translational Medical Research in Cognitive Development and Learning and Memory Disorders, Children’s Hospital of Chongqing Medical University, Chongqing, China; ^2^Wuhan Yiruide Medical Equipment Co., Ltd., Wuhan, China; ^3^Brain Research Center, The University of British Columbia, Vancouver, BC, Canada

**Keywords:** autism spectrum disorders, neonatal isolation, low-frequency repetitive transcranial magnetic stimulation, mIPSCs, GABAA_α1_R, VGAT

## Abstract

Patients with autism spectrum disorder (ASD) display abnormalities in neuronal development, synaptic function and neural circuits. The imbalance of excitatory and inhibitory (E/I) synaptic transmission has been proposed to cause the main behavioral characteristics of ASD. Repetitive transcranial magnetic stimulation (rTMS) can directly or indirectly induce excitability and synaptic plasticity changes in the brain noninvasively. However, whether rTMS can ameliorate autistic-like behaviors in animal model via regulating the balance of E/I synaptic transmission is unknown. By using our recent reported animal model with autistic-like behaviors induced by neonatal isolation (postnatal days 1–9), we found that low-frequency rTMS (LF-rTMS, 1 Hz) treatment for 2 weeks effectively alleviated the acquired autistic-like symptoms, as reflected by an increase in social interaction and decrease in self-grooming, anxiety- and depressive-like behaviors in young adult rats compared to those in untreated animals. Furthermore, the amelioration in autistic-like behavior was accompanied by a restoration of the balance between E/I activity, especially at the level of synaptic transmission and receptors in synaptosomes. These findings indicated that LF-rTMS may alleviate the symptoms of ASD-like behaviors caused by neonatal isolation through regulating the synaptic GABA transmission, suggesting that LF-rTMS may be a potential therapeutic technique to treat ASD.

## Introduction

Autism spectrum disorders (ASDs) are neuropsychiatric developmental diseases characterized by social impairment, repetitive stereotyped behaviors and language retardation (Geschwind, 2011). In recent years, the incidence of ASD has increased sharply around the world. Although frustratingly little is understood about the causal mechanisms underlying this complex disorder, recent studies have suggested that an imbalance of excitatory and inhibitory (E/I) synaptic transmission may cause the main behavioral characteristics of ASD (Gatto and Broadie, 2010).

This hypothesis has been validated in ASD patients (Cornew et al., 2012) and different ASD animal models (Lee et al., 2017). In electroencephalography recordings, ASD children exhibit reduced gamma oscillations in the left hemisphere (Wilson et al., 2007) and elevations in the delta (1–4 Hz), theta (4–8 Hz), alpha (8–12 Hz) and high-frequency (20–120 Hz) power (Cornew et al., 2012). Induction of paired association by using transcranial magnetic stimulation (TMS) in high-functioning ASD patients has proven that there is reduced excitatory synaptic connectivity and deficits in sensorimotor integration (Jung et al., 2013). Based on ASD animal models, an early illuminating review Rubenstein and Merzenich (2003) suggested that an increased E/I (excitation and inhibition) ratio in sensory, mnemonic, social and emotional systems can cause ASD. But the latest review Lee et al. (2017) reminded that the pathogenic mechanisms underlying E/I imbalance in ASD are far more complex than might have been expected.

In a recent review, Uzunova et al. (2016) suggested that repetitive transcranial magnetic stimulation (rTMS) can be applied as a therapeutic modality in ASD to restore the E/I imbalance. rTMS is a technique to provide non-invasive brain stimulation, which consists of the application of rapidly changing magnetic fields to target brain regions by using a coil positioned above the region. The magnetic fields noninvasively penetrate through the intact scalp into the brain and induce electric fields to depolarize neurons. rTMS has been widely used in psychiatry and neurology as well as other clinical specialties since the 1980s (Barker et al., 1985). Recent studies have shown that rTMS can directly or indirectly modulate neuronal excitability and synaptic plasticity in a specific region or the entire brain. For example, high-frequency rTMS (HF-rTMS, 5–20 Hz) induces long-term potentiation, whereas low-frequency rTMS (LF-rTMS, ≤1 Hz) induces long-term depression (Huang et al., 2005). Furthermore, 10-Hz rTMS has been shown to induce a reduction in GABAergic synaptic strength and coordinate Ca^2+^-dependent structural and functional changes of specific inhibitory postsynapses on principal neurons in mice (Lenz et al., 2016). Therefore, rTMS may alleviate autistic behaviors via restoration of the E/I balance.

LF-rTMS has been used to modulate abnormal gamma oscillations resulting from sensory-perceptual deficits in ASD. Twelve sessions of bilateral 1 Hz rTMS to the dorsolateral prefrontal cortex (DLPFC) significantly improved the discriminatory gamma band activity in ASD patients and were associated with improvement in behavioral questionnaire responses (irritability and repetitive behavior; Baruth et al., 2010). In addition, this stimulation also improved error monitoring and correction function in ASD patients (Sokhadze et al., 2012). Additionally, the effects of HF-rTMS on ASD have also been investigated. Two weeks of daily weekday treatment with deep rTMS to the DLPFC (5 Hz, 10-s train duration, 20-s inter-train interval) for 15 min (1500 pulses per session) using a HAUT-Coil on ASD patients yielded a reduction in social-relating impairment and anxiety (Enticott et al., 2014). However, little is known about the cellular and molecular mechanisms underlying the therapeutic effects of rTMS on ASD. More importantly, the different parameters of rTMS (LF-rTMS and HF-rTMS) make understanding the related mechanism more complicated.

While choosing the rTMS parameter to study, we followed the rule that safety is the first priority. HF-rTMS has been reported to occasionally cause side effects such as stroke and seizure (Wassermann, 1998). However, the efficacy, safety, and lack of side effects of LF-rTMS have been validated both in animals (Liebetanz et al., 2003) and in humans (Todd et al., 2006). Furthermore, our previous study showed that LF-rTMS could regulate neural excitability (Tan et al., 2013b). Meanwhile, the potential therapeutic effects of LF-rTMS on ASD patient have been studied and showed that it is helpful to modulate the EEG and abnormal behaviors (Baruth et al., 2010; Sokhadze et al., 2012). Therefore, in the present study, we mainly focused on LF-rTMS and investigated the potential effects of LF-rTMS on autistic-like behaviors in an animal model induced by neonatal isolation. In addition, we further explored the potential mechanisms via electrophysiological and biochemical experiments.

## Materials and Methods

### Animals

This study was carried out in accordance with the recommendations of guidelines for animal research, the Chongqing Science and Technology Commission. The protocol was approved by the Chongqing Medical University Animal Care Committee. Female and male Sprague-Dawley rats were mated (obtained from Chongqing Medical University Animal Care Center) in the laboratory colony of Children’s Hospital of Chongqing Medical University, and the offspring of these pairings served as subjects. Pregnant females were housed individually in plastic cages in the temperature-controlled (21°C) colony room on a 12/12-h light/dark cycle (8 am to 8 pm). Food and water were available *ad libitum*. Within 24 h of birth, all litters were culled to 8–10 pups for each dam. Pups with abnormal body weight (both underweight and overweight) and the redundant ones were culled. Our previous study showed that neonatal isolation induced the same effects both on female and male litters (Wu et al., 2014). Therefore, both genders were used in this study and the sex of the pups was balanced across the whole experiment. The remaining litters were then randomly assigned to either the normally reared control group or the neonatal isolation group.

### Neonatal Isolation

Based on epidemiological studies, adverse childhood experiences (ACEs) have been linked to a host of negative health outcomes, both physical and psychosocial. Numerous ACEs have been associated with long-term behavioral problems (Clarkson Freeman, 2014). Higher ACE scores were significantly associated with all child behavioral subscales (anxiety, depression, aggressive behavior, attention problems, internalizing, externalizing; Fredland et al., 2018). In this regard, neonatal isolation (or maternal separation) is also considered to be a risk factor for neurodevelopmental disorders in children. Exposure of rodents to neonatal isolation has been shown to significantly impair hippocampal neurogenesis (Loi et al., 2014), learning and memory (Marco et al., 2013), as well as social and anxiety behaviors (Tsuda and Ogawa, 2012), including reducing social interaction contact but increasing repetitive and anxiety-like behaviors (Bahi, 2017). In the present study, rats were subjected to neonatal isolation from postnatal day (PND) 1–9 to induce autistic-like behaviors.

The isolation procedure was carried out as described previously (Wu et al., 2014). From PND 1–9, one-half of the pups were designated for isolation. The isolation procedure was performed between 9 am and 12 am each day. During the isolation procedure, each pup was placed approximately 15 cm apart in an individual round plastic chamber (9 cm in diameter, 8 cm in depth, 30°C) without bedding for 1 h. Right after daily isolation, pups were transferred back to the original cage with their dam. After 9 days of isolation, the pups remained with their mothers continuously until weaning on PND 21. Pups of the same treatment condition were then housed with same-sex pups (2–4 within each cage). Control and isolated rats were then randomly separated into two subgroups (LF-rTMS and sham rTMS) on PND 30.

### LF-rTMS Treatment

**Figure 1:**
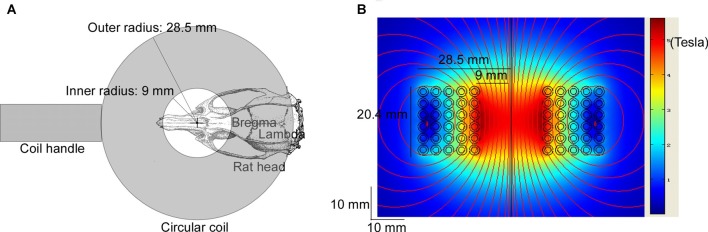
Diagram of repetitive transcranial magnetic stimulation (rTMS) treatment and the modeled induced magnetic field of the circular coil. The Y064 is a parallel-wound solenoidal circular coil (inner radius = 9 mm, outer radius = 28.5 mm, height = 20.4 mm, wire cross-section = 18 mm^2^, number of turns = 5 turns/layer × 6 layers = 30 turns). **(A)** The coil center was placed over the intersection of the midline and the interocular line of the rat (approximately 15 mm anterior to Bregma), which was hand-restrained in a suitable cloth sleeve. The windings of the coil covered an area between 6 mm anterior and 13.5 mm posterior to Bregma. **(B)** The heat map shows the modeled induced magnetic field with 100% of the maximum output of CCY-I, the actual measurement of which was 3.45 Tesla (0 mm below the surface of the circular coil center), 2.50 Tesla (5 mm below) and 1.58 Tesla (10 mm below), respectively.

Rats in the LF-rTMS treatment group (CTR + LF-rTMS and ISO + LF-rTMS) were treated with one session of low-frequency rTMS daily (between 9:00 am and 12:00 am) for 14 consecutive days (PND 30–43). TMS treatment was performed following the previous procedures (Tan et al., 2013a). The rat was hand-restrained in a suitable cloth sleeve. A magnetic-electric stimulator (CCY-I TMS instrument, Yiruide Co., Ltd., Wuhan, China) with a circular coil (Y064, 18-mm inner diameter and 57-mm outer diameter, Figure 1A) was used. The coil is a parallel-wound solenoidal circular coil (height = 2.04 cm, wire cross-section = 18 mm^2^, number of turns = 5 turns/layer × 6 layers = 30 turns, Figure 1B). As a circular coil, the maximal magnetic field is under the center of the coil, but the maximal induced electric field is under the windings of the coil. The coil was placed contiguously with the rat scalp. To stimulate the brain, the coil center was placed over the intersection of the midline and interocular line, which is approximately 15 mm anterior to Bregma. In this situation, the windings of the coil can cover an area between 6 mm anterior and 13.5 mm posterior to Bregma (Figure 1A), the most regions of the rat brain. The pattern of one rTMS session consisted of 20 burst trains, with each train containing 30 pulses at 1 Hz with 2-s inter-train intervals (600 stimuli in total). The stimulation intensity was kept constant in all the experiments and was 100% of the average resting motor threshold of representative animals (as determined by the average stimulation level in 4–5 rats visually inspected for bilateral forelimb movement in a preliminary experiment in awake animals; Gersner et al., 2011), which set to 50% of the maximum output of CCY-I. With this setting, the actual measurement results of the induced magnetic field intensities were 2.60 Tesla (0 mm below the surface of the circular coil center), 1.80 Tesla (5 mm below) and 1.32 Tesla (10 mm below). For the sham rTMS treatment (both sham rTMS CTR and ISO), the coil was turned 90° and kept 5 cm away from the skull.

### Behavioral Tests

After rTMS treatment, the behavioral tests were carried out in the sequence of the three-chamber test, self-grooming test, elevated plus maze test and forced swimming test with 24 h gap between each test. All the behavioral experiments were carried out in dimmed lighting and quiet conditions between 9:00 am and 5:00 pm. Data of three-chamber test and elevated plus maze test were automatically analyzed by ANY-Maze Video Tracking System (Stoelting Co. Wood Dale, IL, USA). Self-grooming and forced swimming scores were firstly recorded with ANY-maze, and then manually scored by a trained person with the same standard double-blindedly. For the double-blinded scoring, all the four groups were randomly coded to A, B, C and D by ANY-maze. It was only allowed to be decoded after all the test had been finished scoring.

### Social Interaction

The social interaction test was performed in a three-chambered apparatus (length × width × height = 60 × 40 × 20 cm). An identical cage was used to enclose a stranger rat (the same gender and age, but different cage) or novel object in each side chamber. And the central chamber was kept empty. Rats were individually tested for 10 min, and the time spent in each chamber was monitored by the ANY-Maze. The ratio of time spent in the rat/object compartment was calculated. The apparatus was then cleaned with 70% ethanol and water between tests.

### Self-Grooming Behavior

Twenty-four hours after habituation, animals were video recorded for 10 min in an open-field arena (60 × 60 cm) and later scored for self-grooming behavior. Self-grooming was defined as licking paws, washing the nose and face or scratching fur with any foot.

### Elevated Plus Maze

For elevated plus maze test, rats were placed in the center of a plus maze that was elevated 1 m above the floor with two opposite open arms and two opposite closed arms (each arm was 50 cm long, the 20-cm-tall walls were only on the closed arms) arranged at right angles. The entries and time spent in the closed and open arms were monitored for 10 min.

### Forced Swimming

In the forced swimming test, rats were placed in a cylinder of water (temperature 24–25°C; 20 cm in diameter, 40 cm in height) for 10 min. The following behaviors were measured: latency to immobility, which was defined as floating or the least movement to maintain the head above the water; and time spent struggling, which was defined as strongly moving all four limbs with the front paws breaking the water surface or scratching the cylinder wall.

### Electrophysiology

The electrophysiology experiment was performed in rats of all the four groups (CTR, CTR + LF-rTMS, ISO and ISO + LF-rTMS). Acute Hippocampal slices were prepared from SD rats (aged 6–7 weeks). Rats were deeply anesthetized by 30% chloral hydrate (3 ml/kg, i.p.) and transcardially perfused with N-methyl-D-glucamine (NMDG) artificial cerebral spinal fluid (ACSF) prior to decapitation. NMDG ACSF contained (in mM): NMDG 93, HCl 93, KCl 2.5, NaH_2_PO_4_ 1.2, CaCl_2_ 0.5, MgSO_4_ 10, NaHCO_3_ 30, HEPES 20, Na-ascorbate 5.0, Na-pyruvate 3.0, Thiourea 2.0, NAC 12, and D-glucose 25, pH = 7.3. Rat brains were rapidly dissected from the skull and placed for sectioning in ice-cold cutting solution (NMDG ACSF) bubbled with 95% O_2_ and 5% CO_2_. Coronal hippocampal slices (400 μm thickness) were sectioned from the middle third of hippocampus with a vibratome (VT1000S, Leica Microsystems, Bannockburn, IL, USA). Slices were then incubated in oxygenated N-2-hydroxyethylpiperazine-N′-2-ethanesulfonic acid (HEPES) ACSF for 1 h at 30°C, which contained (in mM): NaCl 92, KCl 2.5, NaH_2_PO_4_ 1.2, CaCl_2_ 0.5, MgSO_4_ 10, NaHCO_3_ 30, HEPES 20, Na-ascorbate 5.0, Na-pyruvate 3.0, Thiourea 2.0, NAC 12, and 25 D-glucose, pH = 7.3.

Hippocampal CA1 pyramidal neurons were visualized for whole-cell patch clamp recordings under infrared/differential interference contrast microscope (BX51WI, Olympus, Tokyo, Japan). All recordings were conducted at room temperature (about 25°C) using a Multiclamp EPC 10 amplifier (HEKA Electronics, Lambrecht/Pfalz, Germany) and PatchMaster v2.73 software (filtered at 3 kHz and digitized at 10 kHz). For recording miniature excitatory postsynaptic currents (mEPSCs) and miniature inhibitory postsynaptic currents (mIPSCs), cells were held at −70 mV. The external bath solution was standard ACSF (in mM: NaCl 120, KCl 2.5, NaH_2_PO_4_ 1.25, CaCl_2_ 2.0, MgSO_4_ 2.0, NaHCO_3_ 26, glucose 10, pH = 7.3). For mEPSC recording, 1 μM tetrodotoxin (TTX) and 10 μM bicuculline were added into the ACSF. While for mIPSC recording, 1 μM TTX, 20 μM CNQX (6-Cyano-2,3-dihydroxy-7-nitro-quinoxaline) and 50 μM APV (D,L-2-amino-5-phosphonovaleric acid). The patch-pipette internal solution for mEPSC contained (in mM): Cs-methanesulfonate 130, MgCl_2_ 2.0, ethylene-bis (oxyethylenenitrilo) tetraacetic acid (EGTA) 0.5, HEPES 10, QX-314 5.0, K_2_ATP 5.0, and Na_2_GTP 0.3, pH = 7.3. Filled electrodes had resistances of 3–5 MΩ. Moreover, the pipette solution for mIPSC contained (in mM): CsCl 140, CaCl_2_ 0.1, MgCl_2_ 2.0, HEPES 10, EGTA 0.5, and Na_2_ATP 4.0, Na_2_GTP 0.3, QX-314 5.0. mEPSCs and mIPSCs were detected automatically by Mini Analysis Program 6.0.3 (Synaptosoft Inc., Decatur, GA, USA).

### Western Blotting

Anti-GluN1 (ab9864r), anti-GluA2 (mab397), anti-GABAA_α1_R (06868), ant-VGAT (ab5062p) and anti-PSD-95 (mab1598) antibodies were obtained from Millipore. Anti-GluA1 (ab31232) was purchased from Abcam (Cambridge, UK), and anti-β-actin (a5441) antibodies was purchased from sigma.

After behavioral testing, the hippocampi (from both left and right hemisphere) of each animal were collected for Western blotting. The tissue was homogenized in ice-cold Tris-HCl buffer (in mM): 30 Tris-HCl, 4 EDTA, 1 EGTA, 100 (NH_4_)_6_Mo_7_O_24_, 5 Na_4_P_2_O_7_, 25 NaF, 1 Na_3_VO_4_, and a cocktail of protease inhibitors (Complete, Roche), pH = 7.4.

Subcellular fractions were prepared as previously described (Dong et al., 2015). Briefly, the hippocampal homogenates were centrifuged twice at 4°C at 700 *g* for 7 min to remove nuclei and other debris. The two supernatants were pooled and centrifuged at 100,000 *g* at 4°C for 60 min. Pellets were resuspended in the same buffer containing 0.5% Triton X-100 and incubated at 4°C for 20 min, layered over 1 M sucrose, and centrifuged at 100,000 *g* for 60 min. Finally, the Triton-insoluble material that sedimented through the sucrose layer, which is highly enriched in postsynaptic densities, was resuspended in the same buffer containing 1% SDS and stored at −80°C. Total protein concentration was determined by the BCA Protein Assay Kit (Pierce, Rockford, IL, USA).

Samples were aliquoted such that they were in uniform amounts (30 μg for total protein and 10 μg for the subcellular fraction) and boiled with 4× sample buffer at 95°C for 5 min. The samples were then separated on 10% SDS-PAGE gels and transferred onto polyvinylidenedifluoride membranes. To block nonspecific background, the membranes were incubated with 5% fat-free milk for 1 h at room temperature. The target proteins were immunoblotted with primary antibody overnight at 4°C to GluA1 (1:1000), GluA2 (1:500), GluN1(1:3000), VGAT (1:500), and GABAA_α1_R (1:10,000), or followed by incubation with HRP-conjugated secondary antibody (1:3000, 1 h at room temperature). For sequential blotting, the membranes were stripped with stripping buffer and probed with a second antibody. The blots were developed by the Enhanced Chemiluminescence Detection System (Amersham ECL) and imaged by the BioRad ChemiDoc XRS+ system. The intensities of bands of interest (raw data) were quantified using Bio-Rad Quantity One software. The relative level of the target protein is expressed as the percentage difference of the intensity of the target protein and the marker protein, such as postsynaptic marker PSD-95 and cytoplasmic marker β-actin.

### Statistical Analysis

The data were analyzed with GraphPad Prism (GraphPad Software Incorporation, San Diego, CA, USA) and expressed as means ± SEM. All data were analyzed with a two-way analyses of variance (ANOVA) for main effects of animal (CTR or ISO), treatment (LF-rTMS or sham rTMS), and their interaction. All significant effects were further analyzed using Tukey’s *post hoc* test. The significance level was set at *P* < 0.05.

## Results

### LF-rTMS Alleviates Neonatal Isolation-Caused Autistic-Like Behaviors

To determine the effects of LF-rTMS on ASD, we treated the neonatally isolated rats with 1-Hz LF-rTMS for 14 consecutive days. Behavioral tests were then carried out. We selected three behavioral patterns corresponding to those frequently observed in children with autism for examination under laboratory conditions: (1) social interaction deficits, hallmark feature of autism (American Psychiatric Association, 2013); (2) repetitive self-grooming behavior, another core symptom of autism (Bishop and Lahvis, 2011); and (3) anxiety- and depression-related behaviors, which are frequently observed in autistic patients (Strang et al., 2012).

**Figure 2:**
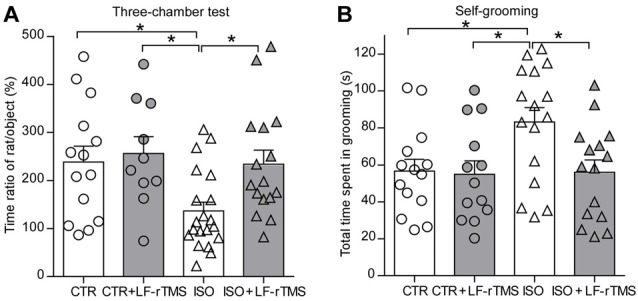
Effects of low frequency (LF)-rTMS on social and repetitive behaviors caused by neonatal isolation. **(A)** Bar graph showing the ratio of time spent with the stranger animal vs. the object compartment in the three-chamber test. **(B)** Self-grooming within 10 min in an open-field arena. CTR: sham rTMS treated control rats without isolation; CTR + LF-rTMS: 14 day’s rTMS treated normal rats; ISO: sham rTMS treated rats with neonatal isolation; ISO + LF-rTMS: isolated rats treated with LF-rTM. Rats used in each group are *n* = 10–20. All data shown are represented as mean ± SEM (*P < 0.05, two-way analysis of variance (ANOVA) and Tukey’s *post hoc* test).

We first tested social interaction in a three-chambered social interaction test (Figure 2A, two-way ANOVA: *F*_animal (1, 56)_ = 4.9, *P* < 0.05; *F*_treatment (1, 56)_ = 4.2, *P* < 0.05; *F*_animal × treatment (1, 56)_ = 2.1, *P* > 0.05). Compared to control normal animals with sham rTMS (CTR), isolated animals with sham rTMS (ISO) displayed a significant decrease in the ratio of time spent with the stranger rat vs. the object compartment (CTR: 239.0 ± 32.4%, *n* = 14; ISO: 136.8 ± 18.3%, *n* = 20; Tukey’s test, *P* < 0.05, CTR vs. ISO). More importantly, this decrease was reversed by LF-rTMS treatment (ISO + LF-rTMS: 234.5 ± 28.5%, *n* = 16; Tukey’s test, *P* < 0.05, ISO + LF-rTMS vs. ISO). These results indicate that neonatal isolation results in autistic-like social deficits and LF-rTMS treatment can overcome it.

Next, we examined the effects of LF-rTMS on the abnormal self-grooming behavior in neonatally isolated rats (Figure 2B, two-way ANOVA: *F*_animal (1, 54)_ = 3.7, *P* = 0.058; *F*_treatment (1, 54)_ = 4.1, *P* < 0.05; *F*_animal × treatment (1, 54)_ = 3.2, *P* > 0.05). Here, we found that animals subjected to neonatal isolation displayed more self-grooming behaviors than control animals (CTR: 56.7 ± 6.4 s, *n* = 14; ISO: 83.2 ± 7.8 s, *n* = 16; Tukey’s test, *P* = 0.047, CTR vs. ISO). Importantly, LF-rTMS treatment restored the excessive self-grooming behavior to the normal level (ISO + LF-rTMS: 56.1 ± 6.7 s, *n* = 15; Tukey’s test, *P* = 0.035, ISO + LF-rTMS vs. ISO). These results suggest that neonatal isolation may also produce autistic-like repetitive behavior, and LF-rTMS treatment can reduce the excessive self-grooming behavior.

**Figure 3:**
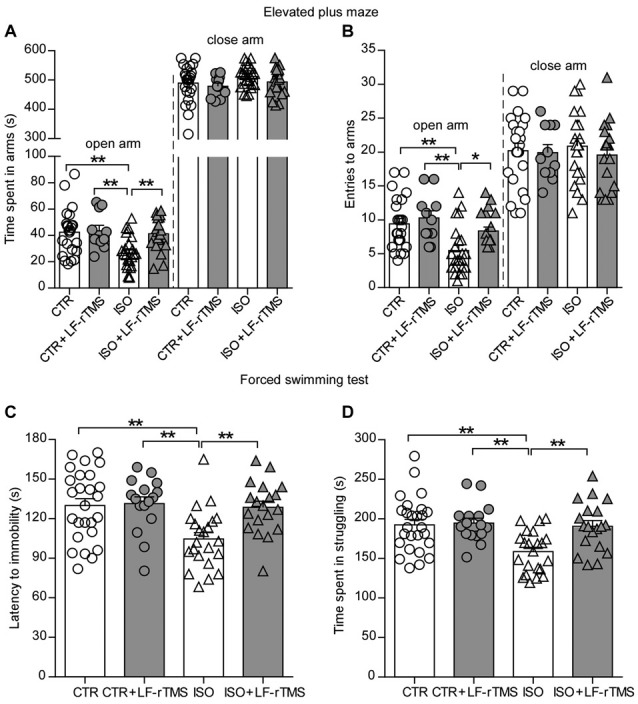
Effects of LF-rTMS on anxiety-/depressive-like behaviors caused by neonatal isolation. Time **(A)** and the number of entries **(B)** into the open and close arms in the elevated-plus maze test. Latency to immobility **(C)** and struggling time **(D)** in the forced swimming test. Rats used in each group are *n* = 11–24. All data shown are represented as mean ± SEM (*P < 0.05, **P < 0.01, two-way ANOVA and Tukey’s *post hoc* test).

Moreover, we introduced different behavioral tests, including the elevated plus maze and forced swimming, to determine the effects of LF-rTMS on the anxiety-/depressive-like behaviors of neonatally isolated animals. Similar to previous reports in patients with autism, rats subjected to neonatal isolation exhibited increased anxiety. Compared to control animals, isolated rats spent significantly less time in the open arms (Figure 3A, two-way ANOVA; *F*_animal (1, 74)_ = 7.2, *P* < 0.01; *F*_treatment (1, 74)_ = 5.4, *P* < 0.05; *F*_animal × treatment (1, 74)_ = 4.1, *P* < 0.05; CTR: 42.5 ± 3.6 s, *n* = 24; ISO: 26.3 ± 2.6 s, *n* = 23; Tukey’s test, *P* < 0.01, CTR vs. ISO) and fewer entries into the open arms (Figure 3B, two-way ANOVA: *F*_animal (1, 74)_ = 12.9, *P* < 0.001; *F*_treatment (1, 74)_ = 5.2, *P* < 0.05; *F*_animal × treatment (1, 74)_ = 1.5, *P* = 0.22; CTR: 9.5 ± 0.8, *n* = 24; ISO: 5.5 ± 0.7, *n* = 23; Tukey’s test, *P* = 0.001, CTR vs. ISO) during the elevated plus maze test. There is no statistically significant differences in both time and entries in the close arms among different groups (Figures 3A,B). Similar to the results observed in the social interaction and self-grooming tests, LF-rTMS treatment was able to restore the open arms time (ISO + LF-rTMS: 41.2 ± 2.9 s, *n* = 20; Tukey’s test, *P* < 0.01, ISO + LF-rTMS vs. ISO; *P* = 0.97, ISO + LF-rTMS vs. CTR) and the number of entries into the open arms (ISO + LF-rTMS: 8.4 ± 0.6, *n* = 20; Tukey’s test, *P* < 0.05, ISO + LF-rTMS vs. ISO; *P* = 0.45, ISO + LF-rTMS vs. CTR) to control levels, indicating that LF-rTMS overcame the neonatal isolation-induced anxiety.

Results from the forced swimming test showed that neonatal isolation reduced both the latency to immobility (Figure 3C, two-way ANOVA; *F*_animal (1, 74)_ = 7.2, *P* < 0.01; *F*_treatment (1, 74)_ = 5.4, *P* < 0.05; *F*_animal × treatment (1, 74)_ = 4.1, *P* < 0.05; CTR: 130.0 ± 5.2 s, *n* = 26; ISO: 104.6 ± 4.8 s, *n* = 23; Tukey’s test, *P* < 0.01, CTR vs. ISO) and the struggling time (Figure 3D, two-way ANOVA; *F*_animal (1, 79)_ = 8.1, *P* < 0.01; *F*_treatment (1, 79)_ = 6.6, *P* < 0.05; *F*_animal × treatment (1, 79)_ = 5.0, *P* < 0.05; CTR: 192.5 ± 6.8 s, *n* = 26; ISO: 158.5 ± 5.5 s, *n* = 22; Tukey’s test, *P* < 0.01, CTR vs. ISO) compared to those in normal rats. In accordance with the anti-depressive effect of rTMS, we found that LF-rTMS treatment obliterated the influence of neonatal isolation on depressive-like behavior (for latency to immobility, ISO + LF-rTMS: 128.7 ± 4.6 s, *n* = 19; Tukey’s test, *P* < 0.01, ISO + LF-rTMS vs. ISO; *P* = 0.99, ISO + LF-rTMS vs. CTR; for struggling time, ISO + LF-rTMS: 190.6 ± 7.2 s, *n* = 19; Tukey’s test, *P* < 0.01, ISO + LF-rTMS vs. ISO; *P* = 0.98, ISO + LF-rTMS vs. CTR).

Taken together, these behavioral results indicate that neonatal isolation causes social deficits and repetitive abnormal behaviors, as well as increased anxiety- and depressive-like behavior. Notably, 14-day LF-rTMS can overcome these abnormal social and emotional alterations.

### LF-rTMS Restores the Neonatal Isolation-Disturbed Inhibitory Synaptic Transmission

**Figure 4:**
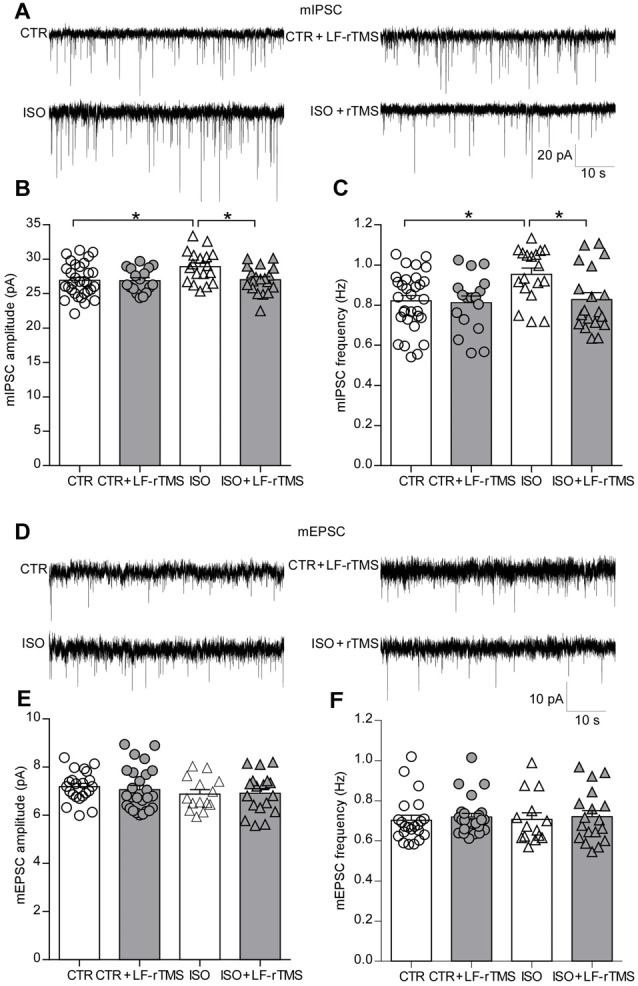
LF-rTMS readjusts the balance between the excitatory and inhibitory synaptic activity of hippocampal pyramidal neurons in neonatally isolated rats.** (A)** Representative miniature inhibitory postsynaptic currents (mIPSCs) traces. Bar graphs of the mIPSC amplitude **(B)** and frequency **(C)**.** (D)** Representative miniature excitatory postsynaptic currents (mEPSCs) traces. Bar graphs of the mEPSC amplitude** (E)** and frequency **(F)**. Cells in each group are 14–31. Rats used are *n* = 5–8 per group. All data shown are represented as mean ± SEM (*P < 0.05, two-way ANOVA and Tukey’s *post hoc* test).

An increase in synaptic inhibition has been reported in genetic animal models of autism, which results in an E/I imbalance (Tabuchi et al., 2007; Pizzarelli and Cherubini, 2013). Accordingly, our previous research also showed that the amplitude of spontaneous inhibitory postsynaptic currents (sIPSCs) significantly increased in CA1 pyramidal neurons of neonatally isolated rats (Wu et al., 2014). To test the effects of LF-rTMS on synaptic transmission, we examined mIPSCs and mEPSCs in hippocampal pyramidal neurons from control and neonatally isolated animals treated with LF-rTMS or sham rTMS. As shown in Figures 4A–C, neonatal isolation significantly upregulated mIPSC amplitude (two-way ANOVA, *F*_animal (1, 81)_ = 4.9, *P* < 0.05, *F*_treatment (1, 81)_ = 4.0, *P* < 0.05, *F*_animal × treatment (1, 81)_ = 3.7, *P* = 0.057; CTR: 26.92 ± 0.43 pA, *n* = 31; ISO: 28.92 ± 0.54 pA, *n* = 18; Tukey’s test, *P* < 0.05, CTR vs. ISO), as well as frequency (two-way ANOVA, *F*_animal (1, 81)_ = 5.3, *P* < 0.05, *F*_treatment (1, 81)_ = 4.5, *P* < 0.05, *F*_animal × treatment (1, 81)_ = 3.3, *P* = 0.073; CTR: 0.82 ± 0.03 Hz, *n* = 31; ISO: 0.95 ± 0.03 Hz, *n* = 18; Tukey’s test, *P* < 0.05, CTR vs. ISO).

Notably, LF-rTMS treatment succeeded in restoring both the mIPSC amplitude (Figure 4B, ISO + LF-rTMS: 27.03 ± 0.45 pA, *n* = 19; Tukey’s test, *P* < 0.05, ISO + LF-rTMS vs. ISO; *P* = 0.10, ISO + LF-rTMS vs. CTR) and frequency (Figure 4C, ISO + LF-rTMS: 0.83 ± 0.04 Hz, *n* = 19; Tukey’s test, *P* < 0.05, ISO + LF-rTMS vs. ISO; *P* = 0.99, ISO + LF-rTMS vs. CTR) to control levels in isolated animals.

However, neonatal isolation did not influence either the mEPSC amplitude (Figure 4E, two-way ANOVA: *F*_animal (1,76)_ = 1.6, *P* > 0.05, *F*_treatment (1,76)_ = 0.06, *P* > 0.05, *F*_animal × treatment (1,76)_ = 0.19, *P* > 0.05; CTR: 7.19 ± 0.13 pA, *n* = 22; ISO: 6.88 ± 0.18 pA, *n* = 14; Tukey’s test, *P* > 0.05, CTR vs. ISO) or mEPSC frequency (Figure 4F, two-way ANOVA, *F*_animal (1,76)_ = 0.01, *P* > 0.05, *F*_treatment (1,76)_ = 0.33, *P* > 0.05, *F*_animal × treatment (1,76)_ = 0.002, *P* > 0.05; CTR: 0.70 ± 0.02 Hz, *n* = 22; ISO: 0.71 ± 0.03 Hz, *n* = 14; Tukey’s test, *P* > 0.05, CTR vs. ISO). Furthermore, LF-rTMS did not affect mEPSC amplitude (ISO + LF-rTMS: 6.91 ± 0.20 pA, *n* = 18; Tukey’s test, *P* > 0.05, ISO + LF-rTMS vs. ISO) or frequency (ISO + LF-rTMS: 0.72 ± 0.03 Hz, *n* = 18; Tukey’s test, *P* > 0.05, ISO + LF-rTMS vs. ISO).

Altogether, these electrophysiological results indicate that neonatal isolation increases only inhibitory but not excitatory synaptic transmission. As expected, LF-rTMS treatment reinstated inhibitory synaptic transmission to the physiological level.

### LF-rTMS Down-Regulates the Increased Expression of Inhibitory Synaptic Receptors Caused by Neonatal Isolation

**Figure 5:**
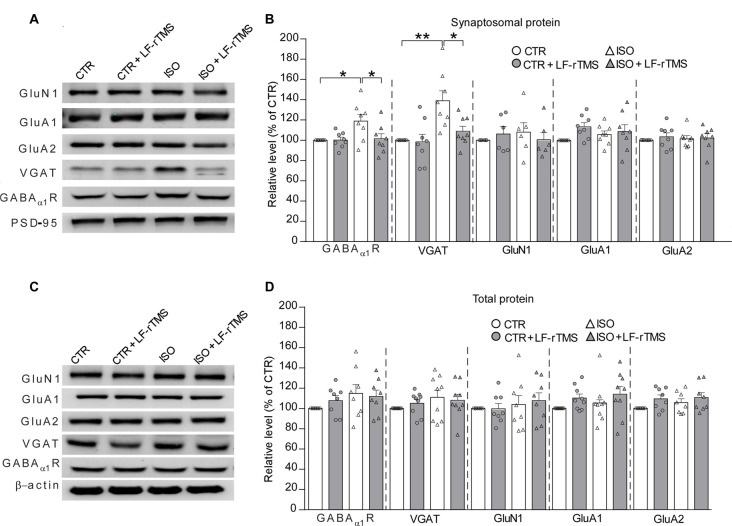
LF-rTMS down-regulates the increased expression of inhibitory synaptic receptors in the hippocampus caused by neonatal isolation. Sequential immunoblotting of synaptosomal fractions **(A,B)** and total tissue lysates **(C,D)** of hippocampal tissues collected from animals after behavioral tests. PSD-95 were used as the postsynaptic marker, and β-actin as the cytoplasmic marker. The relative protein level is normalized by the level of CTR. Rats used are *n* = 7–9 per group. All data shown are represented as mean ± SEM (*P < 0.05, **P < 0.01, two-way ANOVA and Tukey’s *post hoc* test).

Based on the aforementioned electrophysiological experiments which demonstrated that both the amplitude and frequency of mIPSCs increased in rats subjected to neonatal isolation, we next examined whether this change was due to changes in inhibitory receptor and/or transporter expression, including GABAα1R and vesicular GABA transporter (VGAT). Consistent with the electrophysiological results, immunoblot analysis of synaptosomal (synaptic) and lysate (total) inhibitory and excitatory receptors in hippocampal tissues (*n* = 7–9 in each group) revealed a remarkable increase in the postsynaptic expression of GABAα1R (Figure 5B, two-way ANOVA: *F*_animal (1,28)_ = 5.4, *P* < 0.05, *F*_treatment (1,28)_ = 3.9, *P* = 0.057, *F*_animal × treatment (1,28)_ = 4.2, *P* = 0.051; CTR was normalized to 100%, *n* = 8; ISO: 119.0 ± 6.6%, *n* = 8; Tukey’s test, *P* < 0.05, CTR vs. ISO) and VGAT (Figure 5B, two-way ANOVA: *F*_animal (1,28)_ = 13.7, *P* < 0.001, *F*_treatment (1,28)_ = 5.6, *P* < 0.05, *F*_animal × treatment (1,28)_ = 4.5, *P* < 0.05; CTR was normalized to 100%, *n* = 8; ISO: 139.0 ± 9.8%, *n* = 8; Tukey’s test, *P* < 0.01, CTR vs. ISO) in neonatally isolated rats. In comparison, the expression of excitatory synaptic receptor subunits including GluN1, GluA1 and GluA2 remained at the same level (Figure 5B). As expected, LF-rTMS but not sham rTMS was able to downregulate the amount of synaptic GABAα1R and VGAT to normal levels (Figure 5B; for GABAα1R, ISO + LF-rTMS: 101.5 ± 5.0%, *n* = 8; Tukey’s test, *P* < 0.05, ISO + LF-rTMS vs. ISO; *P* = 0.997, ISO + LF-rTMS vs. CTR; for VGAT, ISO + LF-rTMS: 108.9 ± 5.0%, *n* = 8; Tukey’s test, *P* < 0.05, ISO + LF-rTMS vs. ISO; *P* = 0.68, ISO + LF-rTMS vs. CTR). Notably, there was no difference in the total level of either inhibitory or excitatory receptors among all groups (Figure 5D). Original full scans of Western blots were provided in Supplementary Figures S1, S2. These results suggest that neonatal isolation increases GABAAα1R and VGAT content in the hippocampal synaptosomes, which may contribute to the increased inhibitory synaptic neurotransmission, while LF-rTMS reverses the upregulated expression back to the normal level.

## Discussion

In the present study, we confirm that newborn rats subjected to neonatal isolation from PND 1–9 display autistic-like behaviors at the age of young adults and demonstrate that these behavioral alterations are associated with an abnormal increase in inhibitory synaptic transmission and synaptic GABAα1R and VGAT. More importantly, we present results that LF-rTMS treatment for 14 days effectively reduces the increased inhibitory synaptic transmission and synaptic GABAα1R and VGAT, as well as overcomes the autistic-like behaviors caused by neonatal isolation. We have therefore provided evidence that LF-rTMS may restore the balance of E/I synaptic transmission via modulating synaptic receptors and transporters and subsequently ameliorating autistic-like behaviors.

As a noninvasive brain stimulation, rTMS is widely used to treat neurological and psychiatric disorders (Loo and Mitchell, 2005; Spellman et al., 2008), including medication-resistant depression, seizure and schizophrenia. rTMS is well documented to be able to stimulate the nervous system by the electric field induced by the high-intensity magnetic field which is generated from the coil (Barker, 1991). For instance, rTMS is able to depolarize corticospinal neurons either directly at the axon hillock or indirectly via depolarization of interneurons (Pell et al., 2011). Standard TMS with a figure-eight coil (8-coil) has been demonstrated to be able to modulate cortical excitability up to a maximum depth of 1.5–2.5 cm from the scalp (Bersani et al., 2013). As a circular coil, the maximal magnetic field is under the center of the coil, but the maximal induced electric field is under the windings of the coil. To stimulate the brain, especially the cortex and hippocampus, the coil center was placed over the intersection of the midline and interocular line, which is approximately 15 mm anterior to Bregma. As the inner diameter of the coil was 18 mm (9 mm inner radius) and the outer diameter was 57 mm (28.5 mm outer radius), the windings of the coil covered an area between 6 mm anterior and 13.5 mm posterior to Bregma (Figure 1A), covering most regions of the rat brain. The electric fields of 50 coils have been simulated in one study (Deng et al., 2013). The half-value depth (*d*_1/2_) of the electric field penetration has been proven to range from 1.0 cm to 3.5 cm in circular coils. Compared to the size of those coils, the coil used in this study is larger than the animal mini-coil/experimental coil (ID = 16 mm, OD = 26.5 mm, *d*_1/2_ = 0.97 cm) but smaller than the Magstim 50-mm circular coil (P/N 9999, ID = 25 mm, OD = 77 mm, *d*_1/2_ = 1.29 cm). Therefore, the *d*_1/2_ of the coil used in this study may reasonably be assumed to vary from 0.97 cm to 1.29 cm, which means the whole brain of the rat, including the basal regions, is stimulated by the induced electric field. Of course, the maximal induced electric field should be in the superficial cortical regions. For the sham rTMS treatment, the coil was turned 90° and kept 5 cm away from the skull. As the electric field dramatically decreases as the depth increases, the induced electric field would only barely penetrate the brain of the sham rTMS-treated rats.

Many studies have examined the potential brain regions associated with the clinical symptoms of ASD. Specific core regions have been suggested to mediate the clinical phenotypes of ASD including the frontotemporal lobe, frontal-parietal cortex, amygdala, hippocampus, basal ganglia and anterior cingulate cortex (Ha et al., 2015). The frontal cortex is well known to primarily control executive functions, including working memory, decision-making, emotion, social behavior and communication. As the major impairments of ASD are social interaction and emotion, the frontal cortex has received considerable attention in recent years (Donovan and Basson, 2017). The main observations of the frontal cortex in ASD include accelerated total brain volume growth (increased cortical surface area but not thickness) in early childhood followed by a decline in growth and developmental arrest in later childhood that persists into adolescence (Ohta et al., 2016), as well as disorganization of neurons across the cortical layers and connections to other regions. Recent evidence suggests that an abnormality in the hippocampus may also cause some symptoms in ASD. Anatomy research from MRI scans of 24 ASD patients found that the left hippocampus is larger in subjects with ASD than in healthy comparison subjects (Rojas et al., 2006). Abnormal synaptic functions that have been observed in the hippocampus in different ASD mouse models have been summarized in a recent review (Kim et al., 2016). Additionally, the hippocampus connects to several brain regions in the cortical-basal ganglia-thalamic circuits (potential neural pathways implicated in repetitive behaviors), including the striatum and amygdala, suggesting the possibility that the hippocampus may affect repetitive behaviors (Kim et al., 2016). Most interestingly, patients with ASD often have an unusual memory (disrupted recall of everyday events but enhanced ability to recall facts). These traits suggest that the brain’s main memory hub, the hippocampus, may play a critical role in many processes that are clearly absent in ASD, for example, flexible decision making based on past experiences, as well as recall of autobiographical events (one aspect of declarative memory). A preliminary study in animals (Hitti and Siegelbaum, 2014) suggests that disruptions to the hippocampus and its circuits could underlie some of the cognitive difficulties in ASD. Thus, the brain region of primary focus in this study was the hippocampus, as it also plays an important role in the abnormal repetitive behaviors and social memory observed in ASD. Meanwhile, the rTMS-induced electric field can penetrate into the hippocampus in rats. However, for clinical use, non-invasively stimulating the hippocampus by rTMS in a patient is very difficult (only by using specially designed coils). Most importantly, the frontal cortex is a crucial region for ASD; therefore, further research should aim to target cortical regions in the future.

ASD is believed to be a highly heritable disorder, and genetic susceptibility interacts with environmental factors in ASD etiology (Gadad et al., 2013). Although congenital genetic factors are well documented to play a role in 20%–30% of ASD cases, the precise cause of ASD has not been identified for the majority of patients. More and more studies have recently revealed direct or indirect correlations between acquired environmental factors and autism occurrence. For example, valproic acid (Christensen et al., 2013) or lipopolysaccharide (Kirsten et al., 2012) exposure during the critical time period of neural tube closure leads to autistic-like anatomical and behavioral phenotypes in the offspring, suggesting that environmental (non-genetic) factors can contribute to the neuropathophysiology of ASD. Neonatal isolation, a useful model for studying the long-term neurochemical and behavioral changes produced by moderately stressful experiences early in life, can cause autistic-like behaviors in mice and rats (Tsuda and Ogawa, 2012; Wu et al., 2014). Accordingly, in accordance with our previous study (Wu et al., 2014) and some recent studies (Bahi, 2017), we here proved that neonatal isolation can induce ASD-like behaviors including social deficits (Figure 2A), repetitive behaviors (Figure 2B) and anxiety-/depressive-like behaviors (Figure 3) in the offspring.

Previous studies have suggested that an increased E/I (excitation and inhibition) ratio in sensory, mnemonic, social, and emotional systems can cause ASD. This hypothesis is inspired in part by the striking prevalence of seizures among individuals with ASD. Evidence supporting this hypothesis, including fewer GABA receptor subunits has been observed in post-mortem tissue of autistic individuals (Oblak et al., 2011). In addition, the link between GABA and binocular rivalry dynamics is completely and specifically absent in ASD patients (Robertson et al., 2016). Direct optogenetic manipulation of principal excitatory or inhibitory parvalbumin-positive interneurons in the mouse medial prefrontal cortex (mPFC) showed that an elevated (by activating the principal neurons) but not reduced (by activating the inhibitory neurons) E/I balance in the mPFC impairs social behavior (Yizhar et al., 2011). A study in BTBR mice (Han et al., 2014), a model of idiopathic ASD, showed reduced spontaneous GABAergic neurotransmission. In addition, treatment with low doses of benzodiazepines increases inhibitory neurotransmission and improves deficits in social interaction, repetitive behavior and spatial learning. However, a recent report (Lee et al., 2017) has demonstrated that the pathogenic mechanisms underlying the E/I imbalance in ASD are far more complex than might have been expected. More and more data suggest that deviation in synaptic function in either direction may lead to autistic-like behaviors in different kinds of ASD animal models (Kelleher and Bear, 2008). In this study, we observed that neonatal isolation specifically upregulated synaptic GABAα1R and VGAT (Figure 5), which may contribute to the increase in inhibitory synaptic neurotransmission (Figure 4). This is consistent with the hypothesis that the E/I imbalance of synaptic transmission may cause the main behavioral characteristics of ASD. Of course, we should not exclude other cellular and molecular mechanisms underlying the excitation-inhibition imbalance observed in the present work, such as adult neurogenesis (Rizzi et al., 2007; Wu et al., 2014) and epigenetic regulation (Lewis et al., 2013; Marco et al., 2013). Thus, further experiments examining the exact role of neurogenesis and epigenetic regulation will help clarify the basic mechanisms of autistic-like behaviors caused by neonatal isolation. More importantly, we here found that LF-rTMS dramatically decreased the isolation-induced upregulation of synaptic GABAα1R and VGAT (Figure 5) and restored the balance of E/I synaptic neurotransmission (Figure 4) in the hippocampus. However, other brain areas that are also important for emotional and social behaviors, such as the amygdala and prefrontal cortex, should be explored in a future study. Notably, our previous study showed that LF-rTMS increased neural excitability, as reflected by an increase in the APs and sEPSCs of hippocampal CA1 neurons in PND 2–3 pups via affecting the functional properties of VGSCs, A-type K^+^ channels and Ca^2+^ channels (Tan et al., 2013b). However, in the present study, we found that 1-month-old rats subjected to LF-rTMS did not display any increase in the amplitude or frequency of mEPSCs (Figure 4). Discrepancies between these findings still need to be resolved but may be at least in part accounted for by the different animal ages used in these studies. Most importantly, 1-Hz rTMS is generally considered to have an inhibitory effect, whereas, here, we found that it decreased inhibitory transmission. This discrepancy may be due to the different indirect effects of rTMS on various regions. A recent study (Caparelli et al., 2012) reported that brain activation obtained using the simultaneous TMS-fMRI technique before and after application of 1-Hz rTMS over the primary visual-cortex area of individuals who perceived a phosphene revealed that such LF-rTMS suppressed activity in some areas of the brain but increased in other areas. Thus, whether LF-rTMS has the same effect on other brain regions and how LF-rTMS restores the increased inhibitory activity in the hippocampus in neonatally isolated rats need to be studied further in the future.

Trains of rTMS pulses applied at specific frequencies are able to induce persistent modulation of cortical excitability as well as of other physiological, metabolic and behavioral measures (Ziemann, 2004). Thus, rTMS for several consecutive days has been proposed to produce cumulative effects and to be more effective in clinical applications. Indeed, our recent research has proven that 14-day rTMS has more significant effects on neuron excitability and ion channels (Tan et al., 2013b). Accordingly, we reported here that 14-day LF-rTMS alleviated the acquired autistic-like symptoms, including social deficits, repetitive behaviors and anxiety-/depressive-like behaviors (Figures 2, 3). Notably, we only tested the acute effects (1 day after treatment) of rTMS on autistic-like behaviors in neonatally isolated rats in the current study. Further experiments examining the long-lasting influence of LF-rTMS on autistic-like behaviors need to be carried out in the future.

In summary, this study shows that rTMS can ameliorate autistic-like behaviors caused by neonatal isolation in rats by modulating the neuronal E/I balance, indicating that LF-rTMS may be used as a potential noninvasive novel therapeutic method for ASD. However, the stimulation parameters, especially the frequency, intensity and the targeting brain area, of TMS need to be further explored in the future.

## Author Contributions

TT, WW and ZH performed the research. HX provides rTMS technical support and training. TT and ZD designed the research study; contributed essential reagents and tools. TT, WW, YTW and ZD analyzed the data and wrote the manuscript.

## Conflict of Interest Statement

HX is employed by Wuhan Yiruide Medical Equipment Co., Ltd. The other authors declare that the research was conducted in the absence of any commercial or financial relationships that could be construed as a potential conflict of interest.

## Supplementary Material

FIGURE S1 Original full scans of western blots for synaptosomal protein. Related to respective figures as indicated in red frame.

FIGURE S2 Original full scans of western blots for total protein. Related to respective figures as indicated in red frame.
