# Supplementary figures and images for: Low-Frequency rTMS Ameliorates Autistic-Like Behaviors in Rats Induced by Neonatal Isolation Through Regulating the Synaptic GABA Transmission
Source: Front Cell Neurosci. 2018 Feb 28;12:46. doi: 10.3389/fncel.2018.00046 (PMC5835518; doi:10.3389/fncel.2018.00046)

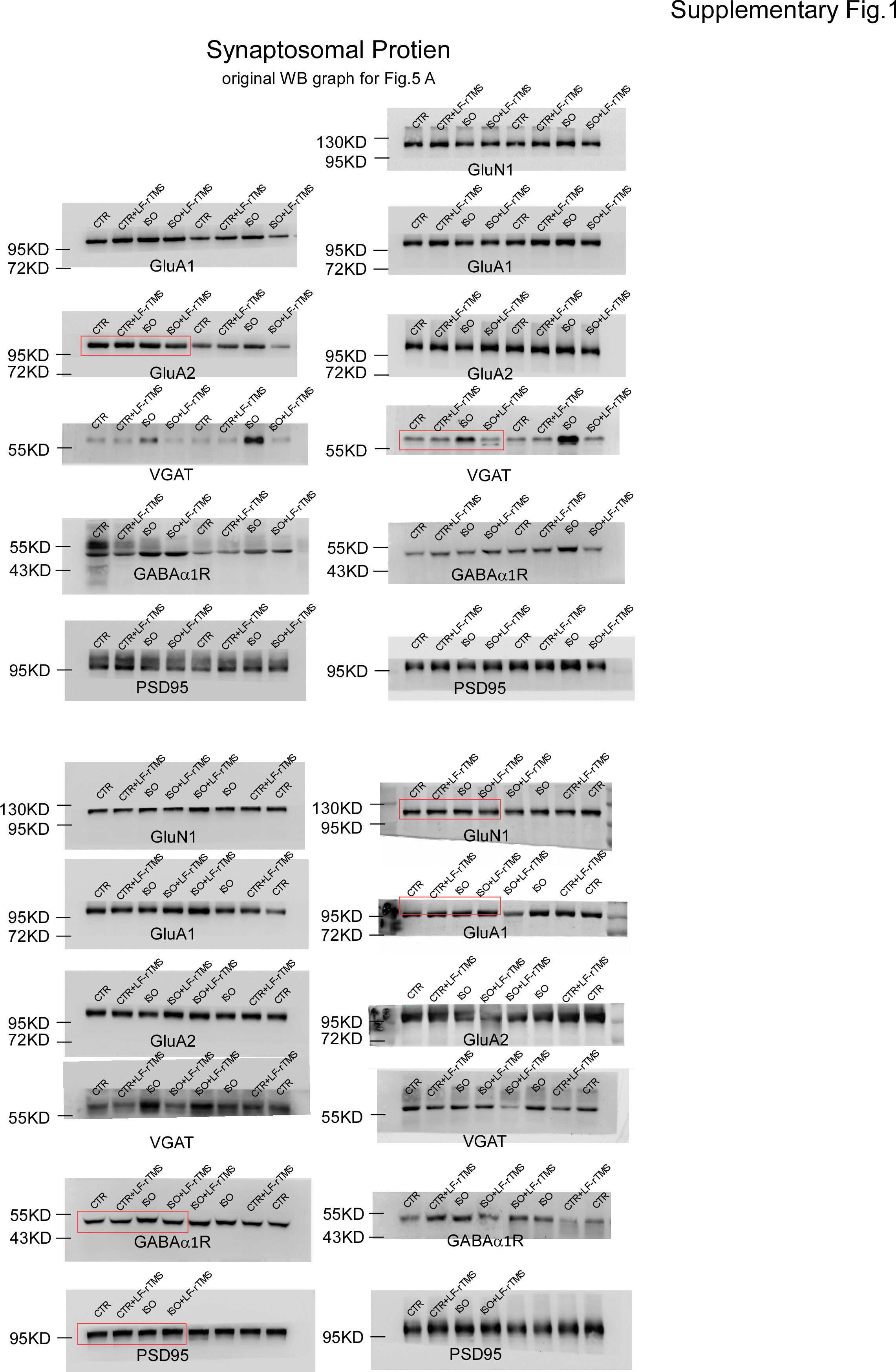

Supplement: FIGURE S1 — Original full scans of western blots for synaptosomal protein. Related to respective figures as indicated in red frame. [file Image_1.jpg]

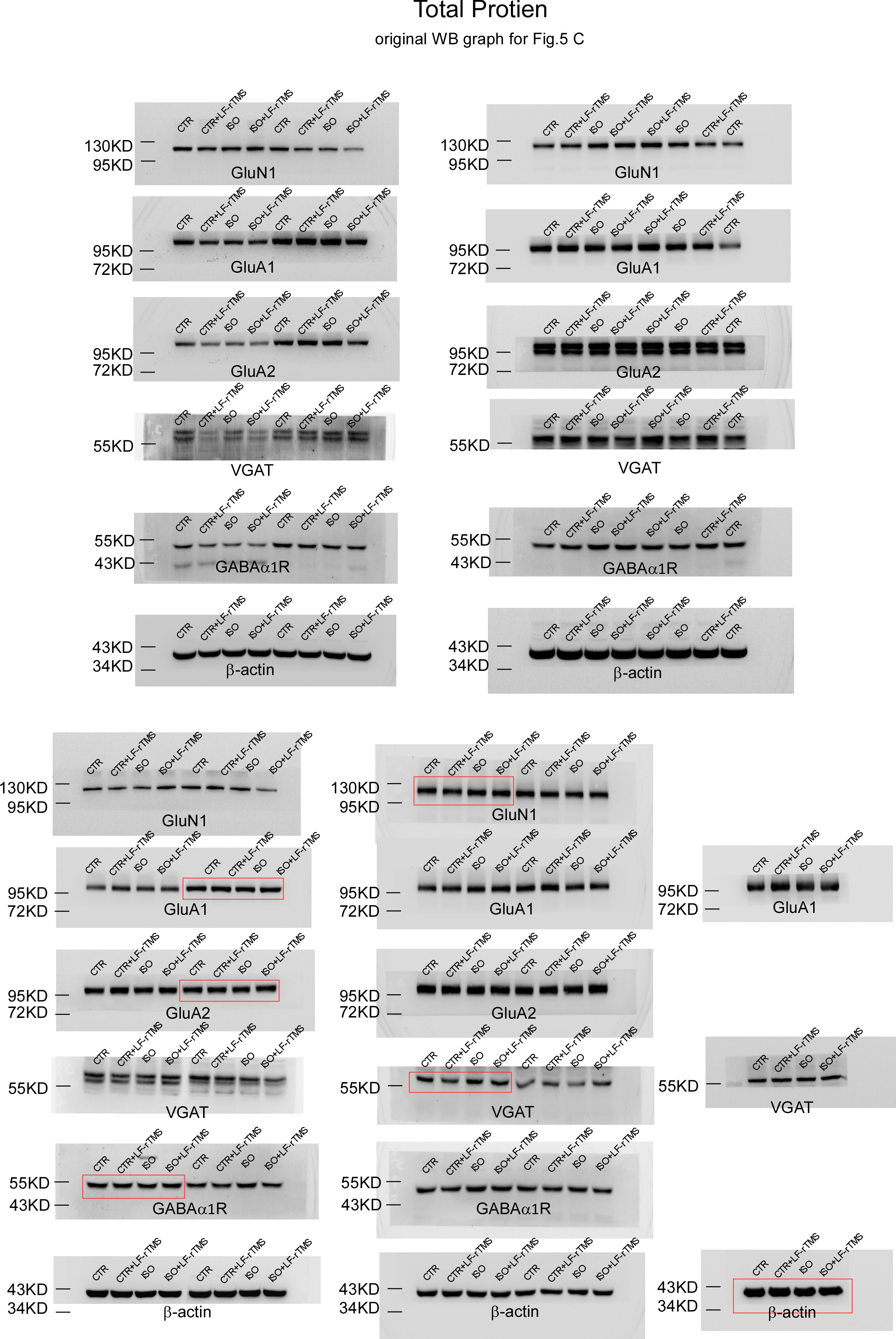

Supplement: FIGURE S2 — Original full scans of western blots for total protein. Related to respective figures as indicated in red frame. [file Image_2.jpeg]
